# Optimal surgical procedure for treating early-stage adenoid cystic carcinoma of the breast

**DOI:** 10.1038/s41598-023-36644-w

**Published:** 2023-06-23

**Authors:** Tao Huang, Qigen Fang, Lianjie Niu, Lina Wang, Xianfu Sun

**Affiliations:** 1 Department of Breast Disease, Henan Breast Cancer Center, The Affiliated Cancer Hospital of Zhengzhou University & Henan Cancer Hospital, Zhengzhou, People’s Republic of China; 2 Department of Head Neck and Thyroid, The Affiliated Cancer Hospital of Zhengzhou University & Henan Cancer Hospital, Zhengzhou, 450008 People’s Republic of China

**Keywords:** Breast cancer, Cancer, Surgical oncology, Cancer

## Abstract

To explore the superiority of breast conservation surgery (BCS) to mastectomy in treating early-stage adenoid cystic carcinoma of the breast (BACC). Patients with surgically treated stage I/II BACC were enrolled between 2000 and 2019 in the SEER database; they were divided into the BCS and mastectomy groups. Overall survival (OS) and disease-specific survival (DSS) were compared between the two groups, and Cox hazard regression models were used to determine the independent predictors. Of the 583 patients in the study, 386 were included in the BCS group. The 10-year OS rates for the BCS and mastectomy groups were 78% (95% CI: 74–82%) and 76% (95% CI: 70–82%), respectively, but the difference was not statistically significant (*p* = 0.968). The 10-year DSS rates for the BCS and mastectomy groups were 95% (95% CI: 93–97%) and 89% (95% CI: 85–93%), respectively, and the difference was statistically significant (*p* = 0.002). Pathological examination of regional lymph nodes and adjuvant treatment were not associated with improved OS or DSS, but age, disease grade, and lymph node metastasis were independent prognostic factors. For stage I/II BACC, BCS can achieve more satisfactory 10-year OS and DSS than mastectomy.

Breast cancer is the most common malignant tumor in women^1^; there were approximately 2.3 million new breast cancer cases and 685,000 breast cancer deaths worldwide in 2020^2^. Of these cases, adenoid cystic carcinoma of the breast (BACC) accounts for less than 0.1%^3^. Most of these tumors exhibit indolent clinical behavior with a low tendency for axillary lymph node involvement and distant metastasis, showing significant differences from other pathologic types. The 10-year survival rate is usually greater than 90%^4^.

Surgery is the mainstay of treatment for BACC owing to the rarity of the disease, related reports are usually case series with limited patients, and the surgical procedures vary from lumpectomy to radical mastectomy^5–9^. The excellent survival profile of patients with BACC raises the important question of whether more breast tissue could be preserved during cancer ablation. Unfortunately, current evidence has only focused on the survival benefit added by adjuvant radiotherapy or chemotherapy, and its sample size is relatively limited^10–15^.

Therefore, the current study aimed to explore the superiority of breast conservation surgery (BCS) to other procedures in treating early-stage BACC.

## Patients and methods

### Patient selection

All data were derived from The Surveillance, Epidemiology, and End Results (SEER) Program, which provides information on cancer statistics to reduce the cancer burden among the US population^16^. The profiles of patients with surgically treated BACC between 2000 and 2019 were retrieved. Patients with stage I/II BACC were included, while those without information on surgical procedures or follow-up were excluded. Data regarding demography, histological grade, tumor size, treatment, and follow-up were extracted and analyzed.

Ethical review was not required as the data were accessible to the public.

### Statistical analysis

The patients were divided into the BCS and mastectomy groups, where BCS referred to partial mastectomy (less than total mastectomy). The Chi-square test was used to assess the differences in clinicopathologic variables between the two groups. The overall survival (OS) and disease-specific survival (DSS) of the groups were compared using the Kaplan–Meier method, and univariate and multivariate analyses were used to detect predictors for OS and DSS in the entire population. All statistical analyses were performed using SPSS 20.0, and p < 0.05 was considered significant.

## Results

### Baseline data

Overall, 583 patients were included in the analysis; of these, 6 were men and 577 were women, with a mean age of 68.5 ± 20.5 years. A total of 386 patients received BCS and 197 patients did not. Both groups had similar distributions of age, sex, marriage, race, side, chemotherapy, and other criteria (all *p* > 0.05). However, 62.1% and 9.1% of patients in the BCS and mastectomy groups, respectively, underwent radiotherapy, and the difference was statistically significant (*p* < 0.001). Patients in the BCS group were more likely to undergo lymph node examination (*p* < 0.001). Compared to 54.3% in the mastectomy group, the BCS group had a lower rate (37.0%) of stage II (*p* < 0.001). Higher pathologic grade was more commonly seen in the mastectomy group (*p* < 0.001) (Table 1).

**Table 1:** Comparison of clinicopathologic variables between breast conservation surgery (BCS) group and mastectomy group.

Variable	Overall cohort (n = 583)	BCS* (n = 386)	Mastectomy (n = 197)	*p*^&^
Age				
≤ 44	47	31	16	
45–59	224	144	80	
60–75	211	140	71	
75 +	101	71	30	0.770
Sex				
Female	577	384	193	
Male	6	2	4	0.187
Marital status				
Married	343	224	119	
Single	71	49	22	
Others	169	113	56	0.816
Race				
White	526	346	180	
Others	57	40	17	0.505
Side				
Left	292	195	97	
Right	291	191	100	0.770
Grade				
I	291	229	62	
II	194	132	62	
III + IV	98	58	40	< 0.001
Radiotherapy	258	240	18	< 0.001
Chemotherapy	73	45	28	0.378
Duration between diagnosis and surgery				
≤ 1 month	477	311	166	
> 1 month	106	75	31	0.274
Regional nodes examined				
0	144	124	20	
1–4	308	224	84	
5–9	77	42	35	
10 +	54	29	25	< 0.001
Disease stage				
I	333	243	90	
II	250	143	107	< 0.001
ER (+)	103	75	28	0.118
PR (+)	55	36	19	0.901
HER2 (+)	3	2	1	1.000

*BCS Breast conservation surgery.

^&^: Comparison between BCS and non-BCS groups.

### Overall survival

**Figure 1:**
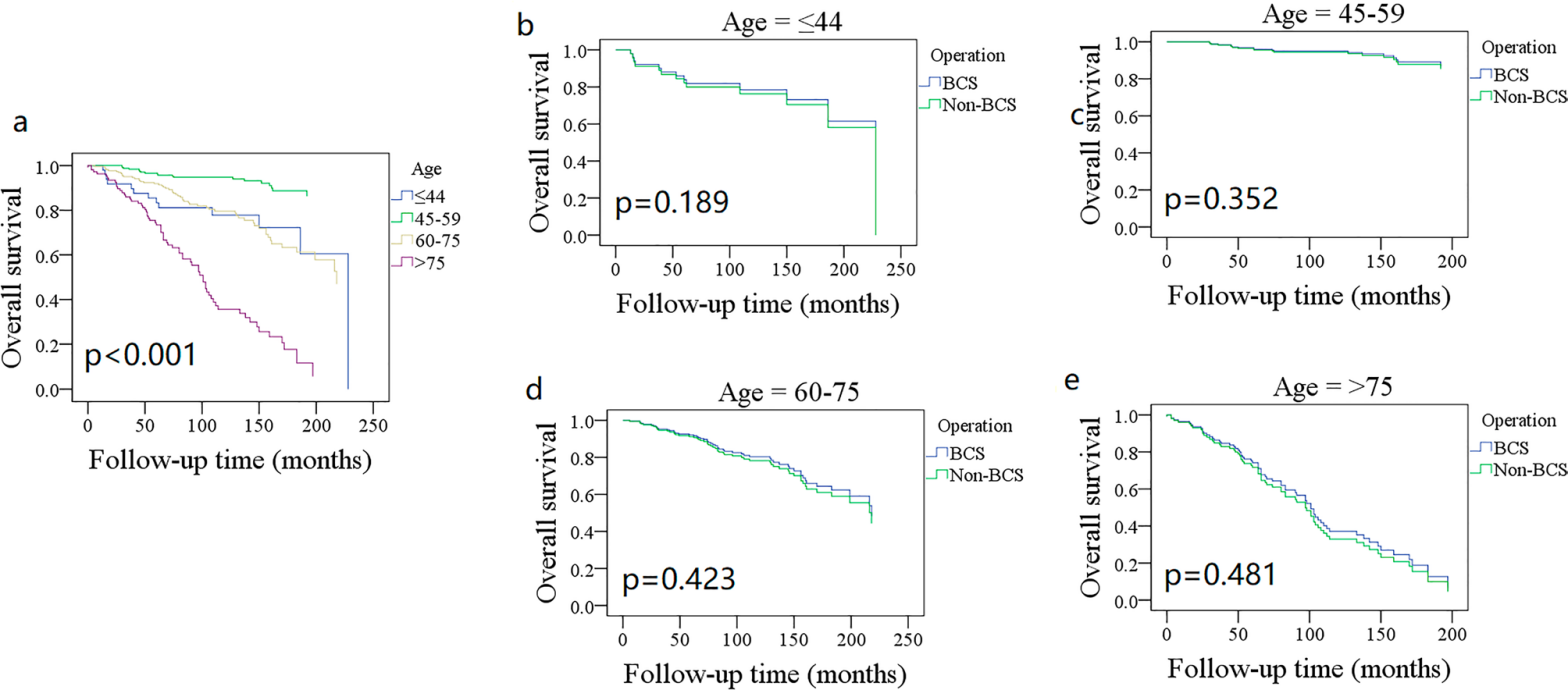
Comparison of overall survival in different age groups.

**Figure 2:**
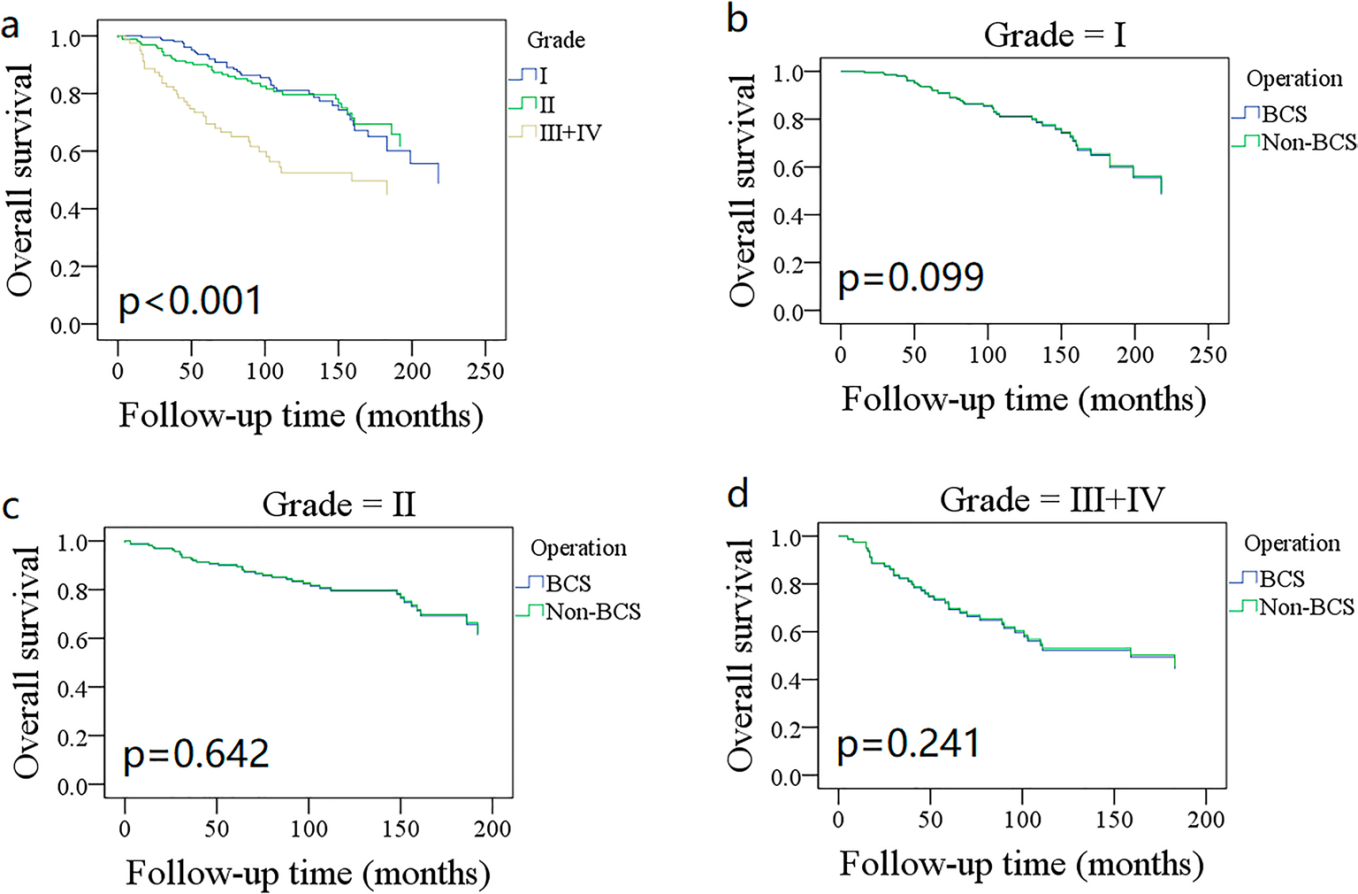
Comparison of overall survival in different grades.

**Figure 3:**
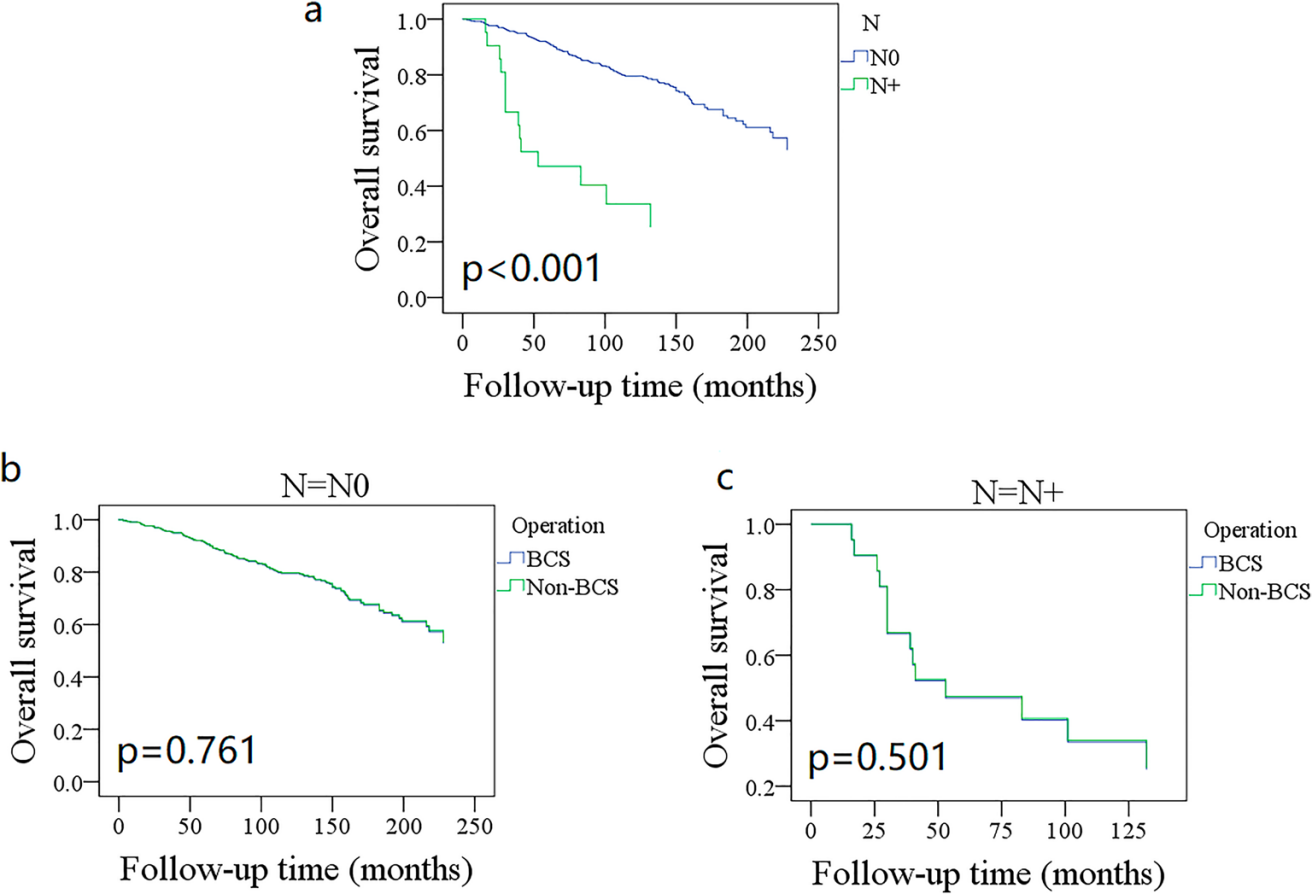
Comparison of overall survival in cases with different regional lymph node statuses.

OS showed different trends among different age subgroups (Fig. 1); patients aged 45–59 years had the best OS with a 10-year rate of 94% (95% CI: 91%–99%), and patients aged more than 75 years had the poorest survival with a 10-year rate of 36% (95% CI: 24–48%). Grade III/IV patients had a 10-year OS rate of 52% (95% CI: 40–64%), which was statistically better than 80% (95% CI: 74–86%) in grade I/II patients (Fig. 2, *p* < 0.001). The 10-year OS rates for the N0 and N + groups were 79% (95% CI: 75–83%) and 34% (95% CI: 12–56%), respectively, and the difference was statistically significant (Fig. 3, *p* < 0.001). Additional Cox model analysis confirmed the independence of patients aged 45–59 years (HR: 0.26; 95% CI: 0.12–0.58), patients aged 75 + years (HR: 2.77; 95% CI: 1.36–5.62), patients with grade III/IV BACC (HR: 1.93; 95% CI: 1.22–3.06), and patients with lymph node metastasis (HR: 4.99; 95% CI: 2.38–10.49) in affecting the OS (Table 2).

**Table 2:** Univariate and multivariate Cox analyses of clinicopathologic variables in affecting the overall survival.

Variable	Univariate		Multivariate	
	*p*	HR [95%CI]	*p*	HR [95%CI]
Age				
≤ 44				
45–59	< 0.001	0.24 [0.12–0.48]	0.001	0.26 [0.12–0.58]
60–75	0.556	0.91 [0.50–1.67]	0.814	0.96 [0.48–1.92]
75 +	< 0.001	3.39 [1.87–6.17]	0.003	2.77 [1.36–5.62]
Sex (Female vs. male)	0.535	1.43 [0.35–5.78]		
Marital status				
Married				
Single	0.204	1.39 [0.84–2.31]	0.317	1.30 [0.73–2.30]
Others	< 0.001	1.92 [1.37–2.70]	0.116	1.40 [0.93–2.11]
Race				
White				
Others	0.563	0.73 [0.10–5.26]		
Side (Left vs. right)	0.388	1.09 [0.80–1.50]		
Operation type (Mastectomy vs. BCS*)	0.968	0.99 [0.71–1.38]		
Grade				
I				
II	0.847	1.01 [0.65–1.56]	0.773	0.97 [0.62–1.52]
III + IV	< 0.001	2.36 [1.52–3.67]	0.002	1.93 [1.22–3.06]
Radiotherapy	0.107	0.78 [0.56–1.07]		
Chemotherapy	0.442	1.16 [0.75–1.78]		
Duration between diagnosis and surgery (≤ 1 month vs. > 1 month)	0.794	0.95 [0.60–1.49]		
Regional nodes examined				
0				
1–4	0.033	0.62 [0.41–0.94]	0.464	0.86 [0.53–1.39]
5–9	0.167	0.64 [0.36–1.12]	0.732	0.92 [0.47–1.80]
10 +	0.662	0.95 [0.57–1.56]	0.358	0.79 [0.42–1.50]
Disease stage (II vs. I)	0.534	1.42 [0.75–2.01]		
N (N + vs. N0)	< 0.001	5.22 [3.00–9.09]	< 0.001	4.99 [2.38–10.49]
ER (Negative vs. positive)	0.333	0.76 [0.48–1.21]		
PR (Negative vs. positive)	0.467	0.82 [0.48–1.43]		
HER2 (Negative vs. positive)	0.309	3.14 [0.44–22.48]		

*BCS breast conservation surgery.

### Subgroup analysis of OS

**Figure 4:**
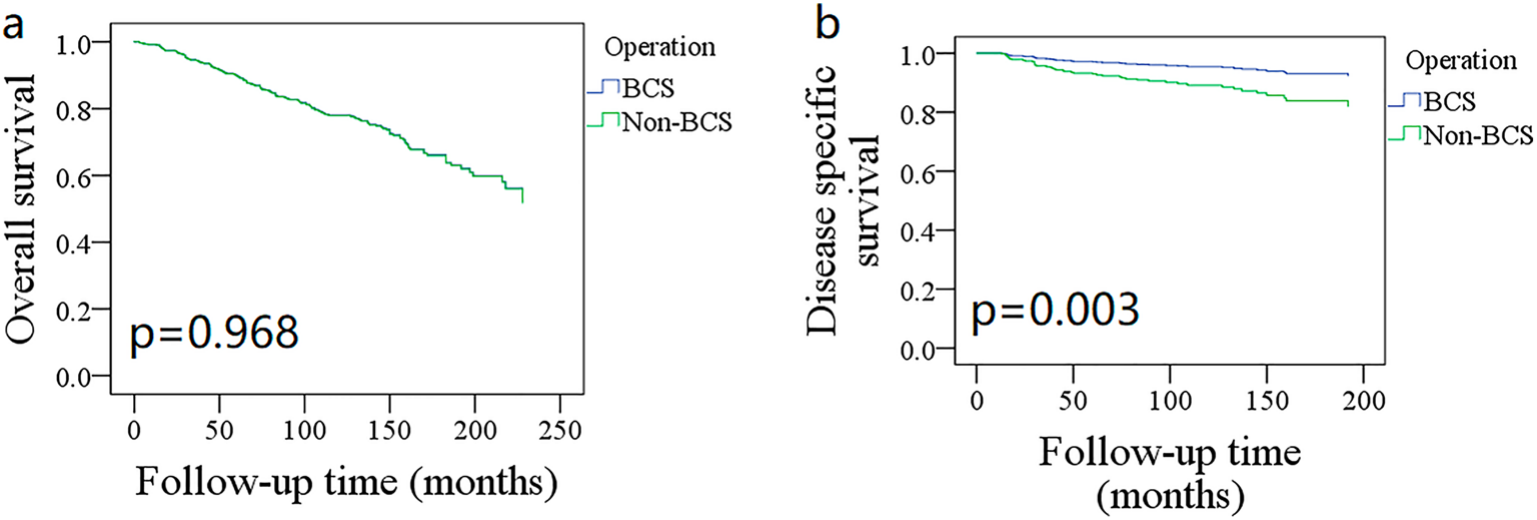
Comparison of overall survival (**A**) disease-specific survival (**B**) between breast conservation surgery (BCS) and mastectomy in the entire cohort.

The 10-year OS rates for the BCS and mastectomy groups were 78% (95% CI: 74–82%) and 76% (95% CI: 70–82%), respectively; the difference was not statistically significant (Fig. 4A, *p* = 0.968). To further explore the potential effect of the type of surgery, OS was compared between different subgroups. BCS and mastectomy patients had a similar 10-year OS distribution for those aged ≤ 44 years (Fig. 1, *p* = 0.189), 45–59 years (Fig. 1, *p* = 0.352), 60–75 years (Fig. 1, *p* = 0.423), and 75 + years (Fig. 1, *p* = 0.481). Grade I (Fig. 2, *p* = 0.099), II (Fig. 2, *p* = 0.642), and III/IV (Fig. 2, *p* = 0.241) patients had comparable OS between BCS and mastectomy. Both N0 and N + groups had an equal distribution of OS (*p* = 0.761 and *p* = 0.501, Fig. 3).

### Disease-specific survival

The 10-year DSS rates for BCS and mastectomy patients were 95% (95% CI: 93–97%) and 89% (95% CI: 85–93%), respectively, and the difference was statistically significant (Fig. 4B, *p* = 0.002). The significance of age, grade, chemotherapy, lymph node metastasis, and HER 2 in influencing DSS was also noted in univariate Cox analysis. Additional multivariate analysis confirmed the independence of age 45–59 years and BCS in improving DSS and that of grade III/IV disease and lymph node metastasis in decreasing DSS (Table 3).

**Table 3:** Univariate and multivariate Cox analyses of clinicopathologic variables in affecting the disease specific survival.

Variable	Univariate		Multivariate	
	*p*	HR [95%CI]	*p*	HR [95%CI]
Age				
≤ 44				
45–59	< 0.001	0.21 [0.09–0.50]	0.007	0.26 [0.10–0.70]
60–75	0.022	0.374 [0.17–0.85]	0.067	0.45 [0.18–1.14]
75 +	0.109	0.50 [0.19–1.29]	0.054	0.08 [0.08–1.09]
Sex (Female vs. male)	0.337	2.34 [0.32–16.98]		
Marital status				
Married				
Single	0.089	2.08 [0.92–4.72]		
Others	0.290	1.73 [0.89–3.33]		
Race				
White				
Others	0.412	2.73 [0.37–19.93]		
Side (Left vs. right)	0.157	1.62 [0.89–2.98]		
Operation type (BCS* vs. mastectomy)	0.002	0.41 [0.22–0.74]	0.012	0.46 [0.23–0.90]
Grade				
I				
II	0.564	1.27 [0.50–3.20]	0.887	1.03 [0.40–2.66]
III + IV	< 0.001	5.59 [2.49–12.54]	0.001	3.84 [1.61–9.14]
Radiotherapy	0.165	0.66 [0.36–1.22]		
Chemotherapy	0.001	2.89 [1.53–5.45]	0.205	1.52 [0.66–3.53]
Duration between diagnosis and surgery (≤ 1 month vs > 1 month)	0.899	0.99 [0.44–2.23]		
Regional nodes examined				
0				
1–4	0.308	1.85 [0.64–5.34]		
5–9	0.111	2.47 [0.74–8.22]		
10 +	0.145	2.53 [0.76–8.44]		
Disease stage (II vs. I)	0.092	1.94 [0.92–5.22]		
N (N + vs. N0)	< 0.001	9.80 [4.52–21.24]	< 0.001	7.57 [2.99–19.21]
ER (Negative vs. positive)	0.357	0.73 [0.31–1.73]		
PR (Negative vs. positive)	0.882	0.83 [0.30–2.32]		
HER2 (Negative vs. positive)	0.049	9.27[1.27–67.86]	0.456	5.17 [0.63–42.52]

*BCS Breast conservation surgery.

## Discussion

This study intended to explore the optimal management of stage I/II BACC, and the most important finding was that BCS could achieve comparable OS to and better DSS than other surgical procedures; pathological examination of regional lymph nodes or adjuvant treatment did not provide an additional survival benefit. This study clarified that BCS alone might be sufficient for treating stage I/II BACC without compromising the prognosis.

There has been considerable progress in breast cancer treatment in recent years, and BCS has become an attractive alternative therapy option for early-stage breast cancer^17^. Saifi et al.^18^ compared OS and DSS between BCS and mastectomy cohorts in stage I/II triple-negative breast cancer using the SEER database and found that BCS patients had 5-year OS and DSS rates of 89% and 93%, respectively, which were both significantly higher than those in mastectomy group. Chu et al.^19^ also reported that the 10-year OS and DSS were better among those who underwent BCS (OS: 80.0%; DSS: 92.7%) than among those who underwent mastectomy (OS: 69.3%; DSS: 88.8%). Even after controlling for co-factors, mastectomy was associated with a 29.8% increased risk of cancer-specific death and a 28.6% increased risk of overall death. van Maaren et al.^20^ reported that the BCS and mastectomy groups showed equal 10-year OS, DSS, and distant metastasis-free survival in T1-2N2 breast cancer, and after stratification, improved OS and DSS were noted in patients with T2N2 disease. Similar findings were also reported in other studies^21,22^. These reports suggest that BCS did not worsen the oncologic prognosis, at least for early-stage breast cancer; however, most BACC cases were triple-negative but had different biological behaviors from classic breast cancer, which then raised the question of whether the same conclusion could apply to BACC. The current study might be the first to report that the two groups showed similar OS, but better DSS with BCS, suggesting the superiority of BCS in treating BACC.

Lymph node metastasis is an adverse pathological feature and predicts a worse prognosis for most cancers^23–25^. The current study supports this viewpoint; lymph node metastasis is related to a nearly fourfold increased risk of overall death and a 6.5-fold increased risk of cancer-caused death. Therefore, timely detection of occult metastasis is important to achieve better regional control. BACC is associated with low lymph node metastasis; no positive lymph nodes were found in six cases in a report by Treitl et al.^6^. Moreover, in a multicenter study of 31 patients who underwent axillary lymph node dissection, only 2 patients had pathological metastases^26^; this is also supported by our findings. Compared to observation, pathological examination of regional lymph nodes did not provide any survival benefit. No similar literature was available for comparison, but we speculated that the finding was contributed to by two factors: one was the low possibility of lymph node metastasis, and another was that the main cause of death was distant metastasis but not regional metastasis^27^.

Owing to the key role of age in prognosis, it was suggested that “age” should be added to a future edition of the AJCC Breast Cancer Staging Manual^28^. A previous study proposed that early-onset breast cancer tended to show adverse features of high grade, triple-negativity, multifocality, and a high proliferation index, translating into worse survival in young patients^29^. Young patients also tended to have a higher number of metastatic sites^30^. However, Berliner et al.’s study found contradicting results^31^. The authors enrolled 174 consecutive patients with breast cancer with central nervous system involvement and divided them into two groups with an age cutoff of 45 years. They reported that the younger group was characterized by longer OS in the entire population and longer DSS in a subgroup of triple-negative breast cancer. All the findings indicated that the role of age remained to be further clarified. In the current study, patients aged 45–59 years had the best OS and DSS among 4 different age subgroups. This finding might be explained by the fact that patients aged 45–59 years had better health status than older patients and tolerated aggressive treatment better. Moreover, as patients aged 45–59 years were usually at the zenith of their careers, economic superiority afforded them better medical resources.

The survival significance of pathologic grade has been widely analyzed. BACC is triple-negative, and triple-negative breast cancer usually carries a poor prognosis and requires systemic chemotherapy. However, BACC is heterogeneous with the presence of transcriptional, marked genetic, clinical, and histologic differences, ranging from low to high grade^32^, histology grade III–IV was related to the hazard of mortality increased, and indicator of adjuvant treatment, although whether adjuvant therapy could optimize the prognosis of patients at high-risk remained a concern^7,8^. However, patients with high-grade cancer have a clinically satisfactory prognosis, which may be explained by the phenomenon found in salivary gland adenoid cystic cancer: distant metastasis was the most common type of treatment failure, but it still maintained a relatively slow growth rate^27^.

Some limitations in the current study must be acknowledged: the SEER database is prone to selection bias. We cannot determine why a patient received nodal sampling, mastectomy, BCS, or adjuvant therapy. Moreover, as data on lymphovascular invasion, margin status, and treatment intent could not be obtained, it might have affected the survival outcome.

In conclusion, for patients with stage I/II BACC, BCS could provide satisfactory 10-year OS and DSS. Pathological examination of regional lymph nodes and adjuvant treatment did not provide an apparent survival benefit; age, disease grade, and lymph node metastasis were the most important prognostic factors.

## Data Availability

All data generated or analyzed during this study are included in this published article. And the primary data could be achieved from the corresponding author.

## References

[1] He J, Chen WQ, Li N, Shen HB, Li J, Wang Y, Li J, Tian JH (2021). Zhou BS; Consulting Group of China Guideline for the Screening and Early Diagnosis and Treatment of Female Breast Cancer; Expert Group of China Guideline for the Screening and Early Diagnosis and Treatment of Female Breast Cancer; Working Group of China Guideline for the Screening and Early Diagnosis and Treatment of Female Breast Cancer [China guideline for the screening and early detection of female breast cancer(2021, Beijing)]. Zhonghua Zhong Liu Za Zhi.

[2] Lei S, Zheng R, Zhang S, Wang S, Chen R, Sun K, Zeng H, Zhou J, Wei W (2021). Global patterns of breast cancer incidence and mortality: A population-based cancer registry data analysis from 2000 to 2020. Cancer Commun..

[3] Ji J, Zhang F, Duan F, Yang H, Hou J, Liu Y, Dai J, Liao Q, Chen X, Liu Q (2022). Distinct clinicopathological and genomic features in solid and basaloid adenoid cystic carcinoma of the breast. Sci. Rep..

[4] Miyai K, Schwartz MR, Divatia MK, Anton RC, Park YW, Ayala AG, Ro JY (2014). Adenoid cystic carcinoma of breast: Recent advances. World J. Clin. Cases..

[5] Kim M, Lee DW, Im J, Suh KJ, Keam B, Moon HG, Im SA, Han W, Park IA, Noh DY (2014). Adenoid cystic carcinoma of the breast: A case series of six patients and literature review. Cancer Res. Treat..

[6] Treitl D, Radkani P, Rizer M, El Hussein S, Paramo JC, Mesko TW (2018). Adenoid cystic carcinoma of the breast, 20 years of experience in a single center with review of literature. Breast Cancer.

[7] Goldbach MM, Hoffman DI, Burkbauer L, Nayak A, Tchou J (2020). Treatment patterns and clinical outcomes of adenoid cystic breast carcinoma: A single-institution experience. Am. Surg..

[8] Zhang M, Liu Y, Yang H, Jin F, Zheng A (2022). Breast adenoid cystic carcinoma: a report of seven cases and literature review. BMC Surg..

[9] Yiğit S, Etit D, Hayrullah L, Atahan MK (2019). Androgen receptor expression in adenoid cystic carcinoma of breast: A subset of seven cases. Eur. J. Breast Health..

[10] Sun JY, Wu SG, Chen SY, Li FY, Lin HX, Chen YX, He ZY (2017). Adjuvant radiation therapy and survival for adenoid cystic carcinoma of the breast. Breast.

[11] Li L, Zhang D, Ma F (2022). Adenoid cystic carcinoma of the breast may be exempt from adjuvant chemotherapy. J. Clin. Med..

[12] Gomez-Seoane A, Davis A, Oyasiji T (2021). Treatment of adenoid cystic carcinoma of the breast: Is postoperative radiation getting its due credit?. Breast.

[13] Zhang D, Li L, Ma F (2022). Prognosis stratification and postoperative radiation therapy utilization in adenoid cystic carcinoma of the breast. Breast.

[14] Yang L, Wang C, Liu M, Wang S (2022). Evaluation of adjuvant treatments for adenoid cystic carcinoma of the breast: A population-based, propensity score matched cohort study from the SEER database. Diagnostics.

[15] Coates JM, Martinez SR, Bold RJ, Chen SL (2010). Adjuvant radiation therapy is associated with improved survival for adenoid cystic carcinoma of the breast. J. Surg. Oncol..

[16] https://seer.cancer.gov/

[17] Margenthaler JA, Dietz JR, Chatterjee A (2021). The landmark series: Breast conservation trials (including oncoplastic breast surgery). Ann. Surg. Oncol..

[18] Saifi O, Chahrour MA, Li Z, Hoballah J, Panoff J, Vallow LA, Zeidan YH (2022). Is breast conservation superior to mastectomy in early stage triple negative breast cancer?. Breast.

[19] Chu QD, Hsieh MC, Lyons JM, Wu XC (2021). 10-year survival after breast-conserving surgery compared with mastectomy in Louisiana women with early-stage breast cancer: A population-based study. J. Am. Coll. Surg..

[20] van Maaren MC, de Munck L, Jobsen JJ, Poortmans P, de Bock GH, Siesling S, Strobbe LJA (2016). Breast-conserving therapy versus mastectomy in T1–2N2 stage breast cancer: A population-based study on 10-year overall, relative, and distant metastasis-free survival in 3071 patients. Breast Cancer Res. Treat..

[21] van Dongen JA, Voogd AC, Fentiman IS, Legrand C, Sylvester RJ, Tong D, van der Schueren E, Helle PA, van Zijl K, Bartelink H (2000). Long-term results of a randomized trial comparing breast-conserving therapy with mastectomy: European Organization for Research and Treatment of Cancer 10801 trial. J. Natl. Cancer Inst..

[22] Kim H, Lee SB, Nam SJ, Lee ES, Park BW, Park HY, Lee HJ, Kim J, Chung Y, Kim HJ, Ko BS, Lee JW, Son BH, Ahn SH (2021). Survival of breast-conserving surgery plus radiotherapy versus total mastectomy in early breast cancer. Ann. Surg. Oncol..

[23] Kawada K, Taketo MM (2011). Significance and mechanism of lymph node metastasis in cancer progression. Cancer Res..

[24] Fang Q, Wu J, Du W, Zhang X (2019). Predictors of distant metastasis in parotid acinic cell carcinoma. BMC Cancer.

[25] Fang Q, Li P, Qi J, Luo R, Chen D, Zhang X (2019). Value of lingual lymph node metastasis in patients with squamous cell carcinoma of the tongue. Laryngoscope.

[26] Wang S, Li W, Wang F, Niu Y, Hao C, Wang X, He L, Tong Z (2017). 36 cases adenoid cystic carcinoma of the breast in China: Comparison with matched grade one invasive ductal carcinoma-not otherwise specified. Pathol Res Pract..

[27] Cantù G (2021). Adenoid cystic carcinoma. An indolent but aggressive tumour. Part B: treatment and prognosis. Acta Otorhinolaryngol. Ital..

[28] Johnson HM, Irish W, Vohra NA, Wong JH (2021). Refining breast cancer prognosis by incorporating age at diagnosis into clinical prognostic staging: Introduction of a novel online calculator. Breast Cancer Res. Treat..

[29] Erić I, Petek Erić A, Kristek J, Koprivčić I, Babić M (2018). Breast cancer in young women: Pathologic and immunohistochemical features. Acta Clin. Croat..

[30] Chen MT, Sun HF, Zhao Y, Fu WY, Yang LP, Gao SP, Li LD, Jiang HL, Jin W (2017). Comparison of patterns and prognosis among distant metastatic breast cancer patients by age groups: A SEER population-based analysis. Sci. Rep..

[31] Ben-Zion Berliner M, Yerushalmi R, Lavie I, Benouaich-Amiel A, Tsoref D, Hendler D, Goldvaser H, Sarfaty M, Rotem O, Ulitsky O, Siegal T, Neiman V, Yust-Katz S (2021). Central nervous system metastases in breast cancer: The impact of age on patterns of development and outcome. Breast Cancer Res. Treat..

[32] Geyer FC, Pareja F, Weigelt B, Rakha E, Ellis IO, Schnitt SJ, Reis-Filho JS (2017). The spectrum of triple-negative breast disease: High- and low-grade lesions. Am J Pathol..

